# Impact of the ‘Healthy Youngsters, Healthy Dads’ program on physical activity and other health behaviours: a randomised controlled trial involving fathers and their preschool-aged children

**DOI:** 10.1186/s12889-022-13424-1

**Published:** 2022-06-10

**Authors:** Philip J. Morgan, Jacqueline A. Grounds, Lee M. Ashton, Clare E. Collins, Alyce T. Barnes, Emma R. Pollock, Stevie-Lee Kennedy, Anna T. Rayward, Kristen L. Saunders, Ryan J. Drew, Myles D. Young

**Affiliations:** 1 School of Education, College of Human and Social Futures, University of Newcastle, Callaghan, NSW 2308 Australia; 2 Active Living Research Program, Hunter Medical Research Institute, New Lambton Heights, NSW 2305 Australia; 3 Centre for Active Living and Learning, College of Human and Social Futures, University of Newcastle, Callaghan, NSW 2308 Australia; 4 College of Health, Medicine and Wellbeing, School of Health Sciences, University of Newcastle, Callaghan, NSW 2308 Australia; 5 Food and Nutrition Research Program, Hunter Medical Research Institute, New Lambton Heights, NSW 2305 Australia; 6 College of Engineering, Science and Environment, School of Environmental and Life Sciences, University of Newcastle, Ourimbah, NSW 2258 Australia; 7 College of Engineering, Science and Environment, School of Psychological Sciences, University of Newcastle, Callaghan, NSW 2308 Australia

**Keywords:** Physical Activity, Fathers, Preschool-aged children, Parenting, Intervention

## Abstract

**Background:**

Targeting fathers may be a key strategy to increase physical activity among their preschool-aged children, but limited research exists in this area. The primary study aim was to examine the impact of a lifestyle program for fathers and their preschool-aged children on child physical activity levels.

**Methods:**

A total of 125 fathers (aged: 38 ± 5.4 years, BMI: 28.1 ± 4.9 kg/m^2^) and 125 preschool-aged children (aged: 3.9 ± 0.8 years, BMI z-score: 0.3 ± 0.9, 39.2% girls) recruited from Newcastle, Australia, NSW were randomised to (i) the Healthy Youngsters, Healthy Dads (HYHD) program, or (ii) wait-list control group. The program included two fathers-only workshops (2 h each) and eight father-child weekly educational and practical sessions (75 min each), plus home-based activities targeting family physical activity and nutrition. Assessments took place at baseline, 10-weeks (post-intervention) and 9-months follow-up. The primary outcome was the children’s mean steps/day at 10-weeks. Secondary outcomes included: co-physical activity, fathers’ physical activity levels and parenting practices for physical activity and screen time behaviours, children’s fundamental movement skill (FMS) proficiency, plus accelerometer based light physical activity (LPA) and moderate-to-vigorous physical activity (MVPA), screen time and adiposity for fathers and children. Process measures included; attendance, satisfaction, fidelity and retention. Linear mixed models estimated the treatment effect at all time-points for all outcomes.

**Results:**

Intention-to-treat analyses revealed a significant group-by-time effect for steps per day at 10-weeks (+ 1417, 95%CI: 449, 2384) and 9-months follow-up (+ 1480, 95%CI: 493, 2467) in intervention children compared to control. There were also favourable group-by-time effects for numerous secondary outcomes including fathers’ physical activity levels, children’s FMS proficiency, and several parenting constructs. No effects were observed for both fathers’ and children’s accelerometer based LPA or MVPA, co-physical activity, screen-time and adiposity measures. Process evaluation data revealed very high levels of satisfaction, attendance, retention, and intervention fidelity.

**Conclusion:**

Engaging fathers in a lifestyle program is a promising strategy to increase physical activity among preschool-aged children. Additional benefits to fathers’ physical activity levels, children’s FMS proficiency and parenting practices further support the importance of engaging fathers to improve family health outcomes.

**Trial Registration:**

Australian New Zealand Clinical Trials Registry: ACTRN12619000105145. Registered 24/01/2019.

**Supplementary Information:**

The online version contains supplementary material available at 10.1186/s12889-022-13424-1.

## Background

Early childhood is a critical time to establish healthy lifestyle behaviour patterns and reduce the risk of later obesity in children [1]. It is a period of rapid physical and cognitive development where children’s habits are formed and the family’s lifestyle habits are open to change [2]. Engagement in physical activity and healthy eating habits in early life is associated with favourable health outcomes, such as improvement to adiposity [3], bone and skeletal health [4], cardio-metabolic health [3, 4], motor skill development [4, 5], psychosocial health [3] and cognitive development [5, 6]. This can result in sustained benefits as lifestyle behaviours developed in early life can persist throughout the life course [7, 8].

Despite this, global estimates suggest that 40 million children under the age of 5 years had overweight or obesity in 2016 [9, 10]. This is likely due to increased engagement in obesity-promoting behaviours, such as physical inactivity [11, 12] and energy-dense, nutrient-poor (EDNP) food consumption [13], which are now commonplace in early childhood (0–5 years of age). In Australia, only 17% of preschool-aged children meet physical activity and screen-time guidelines [11], less than 1% meet the recommended vegetable intake [14] and EDNP foods account for around one third of total energy intake [13].

In response, numerous heathy lifestyle programs have targeted preschool-aged children. A recent meta-analysis of 34 interventions in children aged 0–5 years found a small but significant positive effect for objectively assessed moderate to vigorous physical activity (MVPA), with a mean difference of 2.9 min per day (95%CI: 1.5, 4.2) [15]. However, only 21% of the included interventions were delivered in community/home-based settings and only 32% involved parents. This is a concern as parents’ beliefs, behaviours, and parenting practices have a critical impact on children’s physical activity and other lifestyle behaviours [16, 17]. As such, the review put forth a key recommendation for practitioners and policymakers to focus on changing parent practices to affect change in children's physical activity levels [15].

A criticism of family-based interventions has been the lack of engagement of fathers. Specifically, fathers accounted for just 6% of participating parents from a review of 213 family-based programs that target children’s’ lifestyle behaviours [18]. Despite this, fathers’ play an integral role in promoting health behaviours, especially healthy eating practices [19] and physical activity [20, 21]. A systematic review of 23 studies found fathers’ eating habits to be strongly associated with a child’s dietary intake [19]. This is supported by another review which showed the interactions at mealtimes between fathers’ and children to positively influence children’s long-term eating behaviour [22]. In addition, fathers’ are often more likely to initiate co-participation in physical activity with their children [23, 24] and take part in physical play (e.g., play wrestling) compared with mothers. This physical play often begins in early childhood and the vigorous and stimulating nature of this playstyle can help to improve children’s strength and physical fitness [25]. Furthermore, due to fathers’ increased opportunities and reinforcement to practice sports skills throughout life, they tend to provide a better model of sports skill performance [26–28]. Co-participation in physical activity is a core context for fathers to bond with their children and can lead to a multitude of benefits for children. This includes benefits to physical health, quality of the father-child relationship and children’s’ social-emotional well-being [29, 30].

Given the reported holistic benefits of father-child co-physical activity in early life and the importance of engaging parent’s in their children’s healthy lifestyle behaviours, we developed ‘Healthy Youngsters, Healthy Dads’ (HYHD), the first lifestyle program internationally, that specifically targets fathers and preschool-aged children to improve their physical activity levels. In adhering to the first phase of the Australian Sax Institute’s Translational Research Framework [31], we undertook a feasibility trial of HYHD and demonstrated excellent recruitment, attendance, acceptability, retention, program administration, and promising preliminary intervention outcomes in 24 father/preschool-child dyads [32]. The next phase of the Translational Research Framework is to test the efficacy of the program. Therefore, the primary aim of this randomised controlled trial (RCT) was to test the efficacy of the HYHD program on physical activity (steps/day) of preschool-aged children at the end of the intervention (10-weeks post-baseline). We hypothesised that intervention children would demonstrate significantly greater increases in physical activity at post-intervention (10-weeks) compared to children in the control group. The secondary aim was to test the impact on various secondary outcomes including: (i) days/week participating in co-physical activity, (ii) fathers’ physical activity levels, (iii) fathers’ physical activity and screen time parenting practices, (iv) children’s fundamental movement skill (FMS) proficiency (v) fathers’ and children’s screen-time, (vi) fathers’ and children’s accelerometer based MVPA and (vii) fathers’ and children’s weight status and body composition. The third aim was to test if any impact was sustained at long-term follow-up (9 months post-baseline). The final aim was to assess acceptability of the program through process evaluation (attendance, satisfaction, fidelity and retention). Diet, social-emotional wellbeing and additional parenting outcomes were also collected but will be reported elsewhere.

## Methods

### Study design

The ‘Healthy Youngsters, Healthy Dads’ (HYHD) program was a parallel-group, two-arm Randomised Controlled Trial (RCT) conducted at the University of Newcastle, Australia. In January 2019, family units (fathers and their preschool-aged child) were randomised in a 1:1 ratio to either (i) the HYHD intervention (treatment), or (ii) a waitlist control group. The study received institutional ethics approval (H-2017–0381) and was prospectively registered with the Australian New Zealand Clinical Trials Registry (ACTRN12619000105145). Written informed consent was obtained from all fathers prior to enrolment as well as child assent. The conduct of the study aligned with the CONSORT Statement [33].

### Participants

Between 27^th^ November 2018 to 18^th^ January 2019 families were recruited from the Newcastle region in New South Wales, Australia. The primary recruitment strategies included; a University media release, which featured in several local news outlets (e.g., television, radio and newspaper), distribution of flyers to local early childcare centres, social media posts (Facebook, Instagram and Twitter) and emails to participants of previous University programs. Eligibility criteria for the HYHD program included: were a biological father, step-father, or male guardian of a child aged 3–5 years, lived with their child at least 50% of the week, were able to attend all assessments, indicated availability for program sessions and able to pass a pre-exercise screening questionnaire for physical activity. Fathers who indicated pre-existing health conditions were required to obtain a doctor’s clearance prior to being accepted to the program. Children were eligible for the program if they were of preschool age (3–5 years) and not attending primary school (Kindergarten – Year 6) in the year of the trial. Only one child per participating father could take part in the program [32]. Eligible fathers and children were invited to attend baseline assessments at the University of Newcastle, NSW Australia.

### The HYHD itervention

The 8-week HYHD program supported fathers to optimise their parenting practices in relation to physical activity and nutrition for their preschool-aged children. The components and content were informed by both quantitative and extensive formative qualitative research targeting fathers to improve children’s physical activity and nutrition [25, 34–37]. Core constructs from social cognitive (e.g., self-efficacy, goals, social support) and self-determination (e.g., autonomy, competence, relatedness) theories were incorporated to illicit behaviour change. Also, a full description of intervention components with associated behaviour change techniques and targeted theoretical mediators is provided in Supplementary Table 1 (Additional File 1). Briefly, the intervention comprised three main components; (i) fathers-only workshops, (ii) weekly group sessions for fathers and children and (iii) an Activity Handbook containing weekly home tasks. Both the fathers-only workshops and weekly HYHD sessions were delivered at the University of Newcastle. Four qualified teachers in Physical Education with prior experience in delivering family programs were recruited via email to be facilitators of the HYHD program. Facilitators’ attended training at the University of Newcastle (delivered by PJM). Participants were offered one of three Saturday morning timeslots, delivered by two facilitators. Some facilitators delivered more than one session each week.

(i) *Fathers-only workshops:* Two × 2-h Thursday evening workshops were delivered face-to-face at the University of Newcastle. The first workshop took place a few days before the first session with the children and the second workshop a few days after this. During the workshops, facilitators presented evidenced-based strategies fathers could employ to: i) improve their own lifestyle (physical activity and diet) behaviours, and ii) enhance their parenting practices to improve their children’s physical activity, dietary habits, social-emotional well-being and sports skills. The main topics included: optimising health in the early years, the unique and powerful influence of fathers, SMART goal settings, fundamental movement skills and positive parenting strategies for healthy physical activity, nutrition and screen-time behaviours.
(ii) *Father-child sessions*: Eight × 75-min, weekly group sessions, delivered face-to-face at the University of Newcastle in three separate groups with 20 families per group on Saturdays. Each session was comprised of two components in which fathers and children participated together: (i) a 20-min educational session which alternated weekly topics on physical activity and healthy eating. The weekly themes were: rough and tumble play, vegetables, physical activity, fruit, screens, water and sport skills. As an engagement strategy, each theme was linked to one of several, program animal characters for example, Charlie Chimpanzee (rough and tumble play), and Reg Rhino (Vegetables). (ii) A 55-min practical session including: rough and tumble play (e.g., sock wrestle), FMS practise (e.g., catching, kicking, throwing games) and health-related fitness (e.g. fitness circuits, shuttle carries). To increase family support, mothers and non-enrolled siblings were invited to attend session five and to engage with program resources (including recordings of the fathers-only workshop content) at home and participate in any home-based activities from the Activity Handbook.
(iii) *Home program*: families were encouraged to complete weekly tasks as presented in an Activity Handbook with a choice of activities for fathers and children to complete at home between sessions (approx. 15-min time commitment per week). The activities included: goal setting, FMS practise, physical activity tracking, fathers-only tasks to reinforce positive parenting practise and home challenges matching each session theme (e.g., make a vegetable creature). Families received a Yamax SW200 pedometer to assist with physical activity monitoring. To provide motivation, children earned a weekly animal character sticker if they completed designated home tasks with their father, and a bonus sticker (e.g., banana, basketball) for completing more than one activity.

### Measures

Assessments were held in January (baseline), March (10 weeks, post-intervention) and October (9 months, post-baseline) 2019 at the University of Newcastle, Australia. The primary outcome of the study was the child’s physical activity levels, measured using the average daily step count of seven consecutive days of pedometry (YAMAX SW200 pedometers; Corporation, Kumamoto City, Japan) at 10-weeks. This measure has been validated in preschool-aged children [38, 39] and adults [40]. Participants were asked to wear the pedometer during all waking hours (except when it could get wet or damaged) and to record steps on a log sheet for seven consecutive days. Children were provided with stickers as a motivation to wear their monitors. Daily step count averages were considered a valid recording day and included in the final analysis, if the children had worn the pedometer in the correct position, had completed at least 3 weekdays and 1 weekend day of pedometry, and had reported steps correctly (e.g., reported actual step counts rather than numbers rounded to nearest 1000). Specifically, only one control participant at 10-weeks failed to meet the criteria by not reporting a weekend day, while one intervention participant at 9-months wore the pedometer in an incorrect position and another intervention participant at 9-months incorrectly rounded steps to the nearest thousand. Participants recorded any additional physical activity undertaken, including the duration and intensity, when not wearing the pedometer (e.g., swimming). This was converted to steps using a standardised formula, based on guidelines for children (e.g., 10 min of moderate-to-vigorous intensity physical activity = 1,200 steps) [41]. These additional steps were added to the pedometer step count for an adjusted secondary analysis. Post- intervention assessments were completed in the week after the final session. A detailed description of all other secondary outcomes are provided in Table 1.

**Table 1:** Secondary outcomes measured in ‘Healthy Youngsters, Healthy Dads’ study

Measure	Description
***Fathers and children***	
Physical activity (accelerometer – LPA and MVPA) subgroup of 50 Fathers and children	• For every sequential block of 12 families that complete assessments, 5 were randomly allocated at baseline assessments to complete this measure • One week of wrist-worn accelerometry using wGT3X-BT ActiGraph accelerometers (Actigraph, Pensicola, FL, USA) were used to assess light physical activity (LPA) and moderate-to-vigorous physical activity (MVPA) as average minutes per day. Data were downloaded and analysed using ActiLife version 6.13.4 (Actigraph, Pensacola, FL, USA) **Cut points and minimum wear-time:** • *Preschool-aged children*: Johansson [42] = sedentary ≤ 89 vertical counts (Y) and ≤ 221 vector magnitude (VM) counts per 5 s and ≥ 440 Y counts and ≥ 730 VM counts per 5 s for high-intensity physical activity. Minimum wear-time of 3 days, 7 h/day [43] • *Fathers*: Montoye et al. [44] = VM count cut-points; < 2,860 counts/min (sedentary); 2,860–3,940 counts/min (light); and ≥ 3,941counts/min (moderate-to-vigorous (MVPA). Minimum wear time of 4 days/ 7 h [43]
Father-child co-physical activity	• 2-items adapted from the Youth Media Campaign Longitudinal Survey [45] • Fathers reported on days per week they were physically active with their child one-on-one and with one or more family member
Weight	• Measured in light clothing, without shoes on a digital scale to 0.01 kg (model CH-150kp, A&D Mercury Pty Ltd, Australia) • Weight was recorded at least twice until two measures fell within a range of 0.1 kg, averaged for the analysis
Height	• Measured using the stretch stature method on an electronic stadiometer to 0.1 cm (model BSM370, Biospace, USA) • Height was recorded at least twice until two measures fell within a range of 0.3 cm, averaged for the analysis
BMI	• Calculated using the standard formula, weight (kg)/height in m^2^ • Children’s BMI-z scores were calculated using age- and sex-adjusted standardized scores (z-scores) based upon the UK reference data [46] and LMS methods [47] • International Obesity Task Force cut points were used to determine overweight or obesity [48]
Body composition	• InBody720 bioelectrical impendence analyser, a multi-frequency bioimpedance device (Biospace Co., Ltd, Seoul, Korea) [49]
***Fathers only***	
Physical Activity (Steps/day)	• One week of pedometry using Yamax SW200 pedometers (Yamax Corporation, Kumamoto City, Japan). Validated in adults [40] • Asked to wear all waking hours (except when it could get wet or damaged) and to record steps on a log sheet for seven consecutive days • Daily step count averages were included in the final analysis if they had completed at least 4 days (3 weekdays and 1 weekend day) of pedometry
Self-reported Moderate-to-vigorous physical activity (MVPA)	• Average weekly MVPA measured using modified version of the Godin Leisure Time Exercise Questionnaire [50] • Participants reported average weekly bouts of moderate and vigorous physical activity and average bout length [51]. Values in each category were multiplied and summed to give an overall measure of weekly MVPA
Physical Activity Role Modelling	• Explicit role modelling scale (5-items) from the Activity Support Scale [52] • Internal consistency coefficients has been found to be acceptable for the role modelling subscale among Caucasian parents (α = 0.88) [52]. In the current sample, the internal consistency was: α = 0.85
Screen time	• Adapted version of the Adolescent Sedentary Activity Questionnaire [53] • Fathers reported the total time they spent sitting using screens (of any kind) for anything outside of work on each day in the previous week • This adapted measure has shown good sensitivity to change in previous behaviour change research [36]
Screen time parenting practices	• Assessed with two questionnaires created for the purpose of the study • 1. Screens other than TV represents use of devices other than TV in different contexts (e.g., at a social event, at a restaurant) (total of 7-items). Internal consistency for the current sample was: α = 0.71 • 2. Screens as reward is a single item questionnaire asking fathers if they offered screen based entertainment as a reward for good behaviour
***Children only***	
Object Control Fundamental Movement Skill Competency	• Assessed with seven object control skills described in the validated Test of Gross Motor Development (kick, catch, two-handed and one-handed strike, dribble and overhand and underhand throw [TGMD-3]) [54]) • After watching two live demonstrations, children were filmed performing each skill twice and received a score of 0 or 1 for the presence or absence of various performance criteria (e.g., ball is caught by hands only) • Combined scores for both attempts across all skills represented the overall object control score
Screen time (Mother proxy)	• Adapted version of the Adolescent Sedentary Activity Questionnaire [53] • Mother reported the total time their child spent sitting using screens (of any kind) on each day in the previous week • This adapted measure has shown good sensitivity to change in previous behaviour change research [36]
***Process measures***	
Attendance	• Attendance rate at Fathers-only workshops • Attendance rate across all eight sessions for fathers and children
Program satisfaction	• Process questionnaire developed to determine overall perceptions of program by fathers • Questions were focused on program structure and timing, quality of facilitators, quality of program, quality of program resources (e.g., Activity Handbook), impact of program on behaviour and satisfaction levels • A 5-point Likert scales from 1 (strongly disagree or poor) to 5 (strongly agree or excellent) was used
Fidelity	• Process questionnaire developed for the study to determine overall perceptions of facilitators • Completed by program facilitators • Questions focused on delivery of content for all sessions (e.g., *There was sufficient time to get through all the content*) and perceptions of enjoyment from father and child (e.g., *The youngsters enjoyed the practical session*) • A 5-point Likert scale from 1 (strongly disagree) to 5 (strongly agree) was used • Number and % of practical sessions with all required content delivered. Facilitators were asked to indicate any sessions where they were unable to deliver as intended (e.g., “*If you were unable to complete any rough and tumble activities, please tick the activities you missed below”)*

Demographic information included participant age and fathers’ self-reported employment status, education level, country of birth, ethnicity and marital status. Socioeconomic status was determined using the Australian postal area index of relative socioeconomic advantage and disadvantage [55]. Although assessors were blinded at baseline, this was not achieved for all assessments at follow-up (e.g., participants occasionally wore program shirts to the assessments).

### Sample size

The sample size was based on the primary outcome of the child’s physical activity measured using pedometers. Sixty children in each group was calculated to give the study 80% power to detect a 1,500-step-per-day difference in physical activity change at post-intervention (*p* < 0.05), assuming an attrition rate of 15%. A sample size of 120 children was required, based on a predicted change score standard deviation of 2700 steps/day. These values were derived from step-count change among children who participated in the Healthy Youngsters, Healthy Dads feasibility study [32]. The study was not powered a-priori to detect changes in the secondary outcomes. We did not conduct multiplicity adjustments for these secondary outcomes as they were intended to complement the primary outcome data and provide preliminary insights for definitive hypothesis testing in future studies [56]. In this exploratory context, *p* values < 0.05 for secondary outcomes were interpreted as suggestive, rather than significant effects.

### Randomisation

The randomisation allocation sequences were generated by a statistician using a computer-based random number producing algorithm. Randomisation was stratified by i) a proxy self-reported (father-reported) child physical activity level (above or below median) at baseline assessment [57] and ii) physical activity measurement condition (pedometer only, or pedometer plus accelerometer) to split the sub-sample of participants with accelerometer measured MVPA across the two groups. To note; budgetary constraints meant accelerometer assessments of MVPA were completed on a small sub-sample. After baseline assessments were completed and the data required for stratification was available, all families were randomised after completing baseline assessments. Details of the group assignment were emailed to the family using a standardised template. Complete separation was achieved between the statistician who generated the randomisation sequence, those who concealed allocation and from those involved in implementation of assignments.

### Statistical analysis

All data analyses were conducted using SPSS 26 (IBM Corp., Armonk: NY). All variables were checked for accuracy, missing values and meeting the assumption of normality. Data are presented as mean (SD) for continuous variables and as counts (percentages) for categorical variables. Baseline characteristics for each group were assessed using independent t-tests for continuous variables and chi-squared (χ2) tests for categorical variables. Linear mixed models were used to assess all outcomes for the impact of group (treatment and control), time (treated as categorical with levels baseline, 10 weeks, and 9 months) and the group‐by‐time interaction. Linear mixed models utilise a custom hypothesis test, ensuring adjustment for baseline values in analysis. Analyses included all randomised participants in line with the intention-to-treat principle. Missing data, assumed to be missing at random (MAR), were statistically modelled using a likelihood-based analysis that included all available data. Age, socioeconomic status and sex (child participants only) were examined as covariates to determine whether they contributed significantly to the models. If a covariate was significant, two‐way interactions with time and treatment were also examined and all significant terms were added to the final model. To deal with outliers, standardised values (z scores) were created. Variables which had standardised scores above 3.29 were truncated to a value 1 unit greater than the next lowest value for that variable [58]. Effect sizes were calculated using Cohen d (d = M1-M2/σ pooled). Two sensitivity analyses were also conducted:

1. Completers' analyses for participants who completed all measures at the three assessment time points (baseline, 10 weeks and 9 months).
2. Per‐protocol analyses of HYHD intervention participants who complied well with the assigned treatment compared with control group. ‘Per-protocol’ was defined prior to commencing the trial in the clinical trials registry (ACTRN12619000105145) as those that attended at least 75% of the sessions and completed at least 75% of the home-based tasks (measured by completing an average of 4.5/6 home tasks in the Activity Handbook each week).

## Results

### Participant flow

**Fig. 1:**
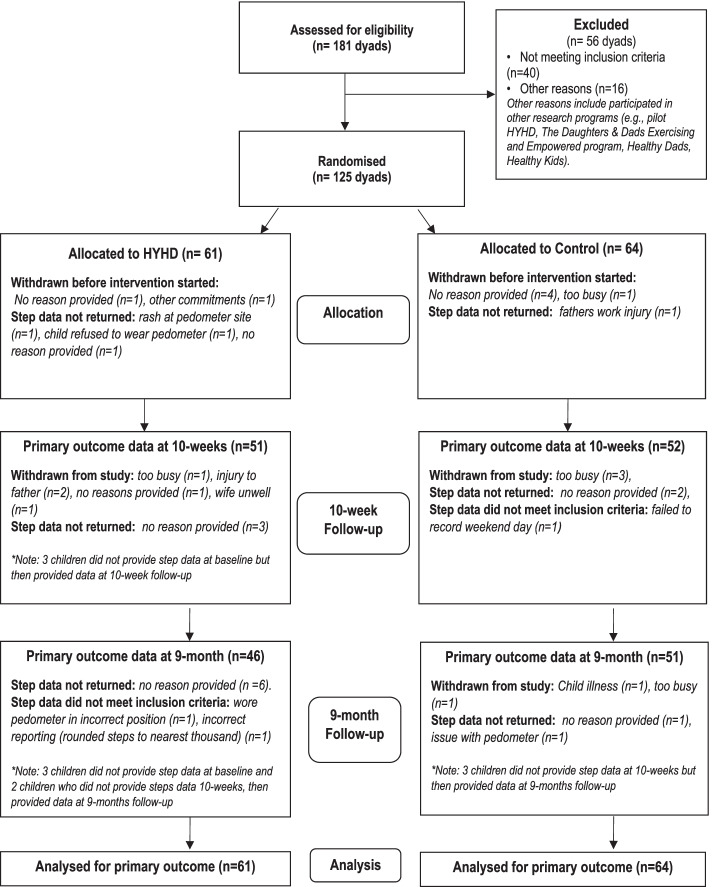
Participant flow through the trial and analysed for primary outcome data (child steps/day)

Figure 1 illustrates the flow of participants through the trial. A total of 181 fathers were assessed for eligibility. In total, 125 fathers and their children completed baseline assessments and were randomised by family unit. Overall, 88% of the dyads were retained at 10 weeks post baseline assessments (*n* = 110) and 87% at 9 months follow-up (*n* = 109). Follow-up data were obtained for the primary outcome (pedometer steps in children) from 82% of children at 10 weeks post baseline assessments (*n* = 103) and 78% at 9 months (*n* = 97). Fathers and children who did not return for follow-up assessments were not significantly different to those who returned for most demographic variables or baseline study outcomes (*p* > 0.05). The only exception was a greater reported baseline screen time use among fathers who returned versus those who did not return at 9-months (*p* < 0.001). For the accelerometer based sub-sample for LPA and MVPA, fathers were required to reach at least 10 h of valid wear time on at least 4 days per week, while children were required to reach at least 7 h of valid wear time on at least 3 days per week. At baseline, this threshold was met by 43 fathers (86%) and 42 children (84%). At 10-weeks 46 of the 50 families provided accelerometer data and of these 37 fathers (80%) and 39 children (85%) met the wear-time requirements. At 9-months 42 of the 50 families provided accelerometer data and of these 33 fathers (79%) and 35 children (83%) met the wear-time requirements.

### Baseline data

The baseline characteristics of the fathers and children are presented in Table 2. Fathers’ mean (SD) age was 38.0 years (5.4) and mean BMI was 28.1 (4.9). Overall, 33% of the fathers were living with obesity (BMI ≥ 30 kg/m^2^). The mean (SD) age of children was 3.9 (0.5) years, 61% were boys and mean BMI z-score was 0.32 (0.89), with 26% of the sample at risk of becoming overweight. The average daily step counts at baseline were 8263 (2913) and 8837 (2653) for fathers and their children respectively.

**Table 2:** Demographic characteristics of study participants

**Child**	**Control** (*n* = 64)		**HYHD** (*n* = 61)		**Total** (*n* = 125)	
	**Mean**	**SD**	**Mean**	**SD**	**Mean**	**SD**
Age (y) (*n* = 125)	3.9	0.5	4.0	0.5	3.9	0.5
Weight (kg) (*n* = 124)	17.2	2.3	17.7	2.4	17.5	2.4
Height (cm) (*n* = 124)	103.3	6.3	104.0	5.5	103.6	5.9
Body fat mass (%) (*n* = 121)	17.6	5.7	17.8	7.7	17.7	6.7
Body Mass Index (kg/m^2^) (*n* = 124)	16.1	1.1	16.3	1.4	16.2	1.3
Body Mass Index z-score (*n* = 124)	0.2	0.8	0.4	1.0	0.3	0.9
Physical activity (steps per day) (*n* = 114)	9595.9	2596.6	8051.6	2499.2	8837.3	2653.7
	**n**	**%**	**n**	**%**	**n**	**%**
Sex						
*Male*	42	65.6%	34	55.7%	76	60.8%
Body Mass Index z-score category ^a^ (*n* = 124)						
*Healthy weight (-2.0 to 1.0)*	50	79.4%	41	67.2%	91	73.3%
*Risk of overweight (*> *1.0)*	13	20.6%	19	31.1%	32	25.8%
*Obesity (*> *3.0)*	0	0.0%	1	1.6%	1	0.8%
**Fathers**	**Control (*n* = 64)**		**HYHD (*n* = 61)**		**Total (*n* = 125)**	
	**Mean**	**SD**	**Mean**	**SD**	**Mean**	**SD**
Age (y) (*n* = 125)	38.4	4.9	37.6	5.9	38.0	5.4
Weight (kg) (*n* = 125)	90.9	17.3	90.9	19.5	90.9	18.3
Height (cm) (*n* = 125)	179.5	7.5	179.6	7.3	179.6	7.4
Body fat mass (%) (*n* = 124)	23.1	8.5	22.3	8.3	22.7	8.3
Body Mass Index (kg/m^2^) (*n* = 125)	28.2	4.8	28.1	5.1	28.1	4.9
Physical activity (steps per day) (*n* = 117)	8160.2	2906.3	8368.2	2906.3	8263.3	2913.8
	**n**	**%**	**n**	**%**	**n**	**%**
Education level (*n* = 125)						
*Post-school qualifications*	59	92.2%	56	91.8%	115	92.0%
Employment status (*n* = 125)						
*Full-time*	57	89.1%	55	90.2%	112	89.6%
Currently attending an education institution (*n* = 125)						
*Full-time or Part time*	6	9.4%	5	8.2%	11	8.8%
*Not a student*	58	90.6%	56	91.8%	114	91.2%
Aboriginal or Torres Strait Islander (*n* = 125)	3	4.7%	1	1.6%	4	3.2%
Born in Australia (*n* = 125)	56	87.5%	53	86.9%	109	87.2%
Relationship status (*n* = 125)						
*Single*	0	0.0%	2	3.3%	2	1.6%
*Married/defacto*	63	98.4%	59	96.7%	122	97.6%
*Separated*	1	1.6%	0	0.0%	1	0.8%
Body Mass Index category (*n* = 124)						
*Underweight (*< *18.5 kg/m*^*2*^*)*	1	1.6%	0	0.0%	1	0.8%
*Healthy weight (18.5 to 24.9 kg/m*^*2*^*)*	15	23.8%	16	26.2%	31	25.0%
*Overweight (25 to 29.9 kg/m*^*2*^*)*	29	46.0%	22	36.1%	51	41.1%
*Obesity (*≥ *30 kg/m*^*2*^*)*	19	30.2%	22	36.1%	41	33.1%
Socio-economic status ^b^ (*n* = 125)						
*1–2 (lowest)*	1	1.6%	1	1.6%	2	1.6%
*3–4*	16	25.0%	18	29.5%	34	27.2%
*5–6*	26	40.6%	22	36.1%	48	38.4%
*7–8*	16	25.0%	18	29.5%	34	27.2%
*9–10 (highest)*	5	7.8%	2	3.3%	7	5.6%

^a^BMI-z calculated using the LMS method (World Health Organization growth reference centiles) [59]. ^b^Socio-economic status by population decile for SEIFA Index of Relative Socio-economic Advantage and Disadvantage[60]

### Primary outcome

As outlined in Table 3, children’s mean physical activity levels significantly increased by 1895 steps/day in the HYHD group at 10 weeks (post-intervention), compared with 478 steps/day in the control group (difference between groups = 1417 steps/day, 95% CI: 449 to 2384, *d* = 0.5). The significant effect was sustained at 9-months (difference between groups = 1480 steps/day, 95% CI: 493 to 2467, *d* = 0.6). In addition, results were consistent with those produced in both the completers and per-protocol analyses (see Supplementary Tables 2 and 3 in Additional File 1).

**Table 3:** Changes in primary and secondary outcomes for study participants (intention-to-treat)

Outcome	Group	Baseline	10 weeks change from baseline (Mean, 95% CI)			9 months change from baseline (Mean, 95% CI)		
		**Mean (SE)**	**Within group**^**a**^	**Mean difference between groups**^**b**^	***p*-value [Cohen’s *d*]**	**Within group**^**c**^	**Mean difference between groups**^**b**^	***p*-value [Cohen’s *d*]**
Primary Outcome								
Steps/day								
Children ^e, h^	Intervention	8043 (342)	** + 1895 (1202, 2588)**	** + 1417 (449, 2384)**	**.004 [0.53]**	** + 1996 (1281, 2712)**	** + 1480 (493, 2467)**	**.003 [0.55]**
	Control	9596 (339)	+ 478 (-197, 1154)			+ 516 (-164, 1198)		
Secondary Outcomes								
Steps/day								
Father ^e^	Intervention	8368 (379)	** + 850 (229, 1470)**	** + 1027 (157, 1897)**	**.021 [0.43]**	** + 737 (97, 1376)**	+ 633 (-254, 1520)	.161 [0.26]
	Control	8160 (377)	-177 (-788, 433)			+ 104 (-511, 718)		
Adjusted steps/day^i^								
Children ^e,h^	Intervention	10,625 (498)	+ 406 (-571, 1385)	** + 1500 (135, 2865)**	**0.032 [0.40]**	+ 1122 (-28, 2271)	** + 1904 (306, 3503)**	**0.020 [0.44]**
	Control	12,095 (492)	**-1093 (-2045, -141)**			-783 (-1894, 329)		
Fathers ^e^	Intervention	10,104 (479)	+ 217 (-676, 1109)	+ 1040 (-212, 2292**)**	0.103 [0.29]	+ 371 (-548, 1290)	+ 1046 (-230, 2322)	0.108 [0.30]
	Control	9824 (477)	-823 (-1702, 56)			-675 (-1560, 210)		
LPA (accelerometer sub-sample) (mins/d)								
Children (*n* = 43)^j^	Intervention	249 (7)	-4 (-20, 11)	-3 (-24, 19)	0.798 [-0.07]	+ 3 (-13, 20)	-6 (-28, 16)	0.602 [-0.16]
	Control	246 (7)	-2 (-17, 13)			+ 9 (-6, 24)		
Fathers (*n* = 45)^k^	Intervention	174 (10)	**-54 (-78, -31)**	-10 (-43, 22)	0.516 [-0.19]	-9 (-35, 16)	-4 (-38, 30)	0.823 [-0.07]
	Control	186 (10)	**-44 (-66. -22)**			-5 (-28, 17)		
MVPA (accelerometer sub-sample) (mins/d)								
Children (*n* = 43)^j^	Intervention	104 (6)	+ 5 (-8, 18)	-4 (-22, 14)	0.636 [-0.15]	** + 22 (8, 36)**	+ 2 (-17, 21)	0.823 [0.07]
	Control	108 (6)	+ 9 (-3, 22)			** + 20 (7, 32)**		
Fathers (*n* = 45)^k^	Intervention	70 (10)	** + 51 (14, 89)**	+ 11 (-40, 62)	0.665 [0.13]	+ 1 (-19, 21)	+ 6 (-20, 33)	0.630 [0.14]
	Control	90 (11)	** + 40 (6, 75)**			-5 (-23, 12)		
Self-reported MVPA (mins/wk)								
Fathers ^e^	Intervention	140 (26)	** + 61 (17, 105)**	+ 60 (-1, 122)	.055 [0.35]	+ 34 (-20, 85)	+ 57 (-17, 130)	.132 [0.27]
	Control	174 (25)	+ 1.0 (-43, 44)			-24 (-76, 28)		
Children’s FMS competence (TGMD)								
Object control score ^d, f, h^	Intervention	8.9 (0.6)	** + 4.7 (3.3, 6.2)**	** + 4.5 (2.5, 6.5)**	**.000 [0.79]**	** + 7.9 (6.4, 9.3)**	** + 2.7 (0.6, 4.8)**	**.011 [0.46]**
	Control	10.6 (0.6)	+ 0.3 (-1.1, 1.7)			** + 5.1 (3.7, 6.6)**		
Co-physical activity (days/wk)								
1-on-1	Intervention	1.6 (0.2)	** + 0.9 (0.4, 1.3)**	+ 0.4 (-0.3, 1.1)	.252 [0.21]	+ 0.4 (-0.1, 0.9)	+ 0.2 (-0.5, 0.9)	.665 [0.08]
	Control	1.3 (0.2)	+ 0.5 (-0.0, 1.0)			+ 0.2 (-0.3, 0.7)		
Family (other children or family)	Intervention	2.5 (0.2)	+ 0.3 (-0.2, 0.8)	+ 0.2 (-0.4, 0.9)	.470 [0.13]	+ 0.03 (-0.4, 0.5)	+ 0.05 (-0.6, 0.7)	.879 [0.03]
	Control	2.3 (0.2)	+ 0.1 (-0.4, 0.5)			-0.02 (-0.5, 0.5)		
Fathers’ role modelling								
Physical Activity	Intervention	2.7 (0.1)	** + 0.4 (0.2, 0.5)**	** + 0.3 (0.1, 0.5)**	**.001 [0.58]**	** + 0.3 (0.2, 0.5)**	** + 0.3 (0.1, 0.5)**	**.003 [0.54]**
	Control	2.7 (0.1)	+ 0.1 (-0.0, 0.2)			+ 0.0 (-0.1, 0.2)		
Screen time parenting practices								
Fathers’ screen as reward ^e, f^	Intervention	2.4 (0.1)	**-0.6 (-0.7, -0.4)**	**-0.3 (-0.6, -0.1)**	**.007 [0.49]**	**-0.6 (-0.8, -0.4)**	**-0.3 (-0.6, -0.1)**	**.011 [0.46]**
	Control	2.3 (0.1)	**-0.3 (-0.4, -0.1)**			**-0.3 (-0.5, -0.1)**		
Fathers’ screens other than TV ^f, g^	Intervention	1.6 (0.7)	**-0.4 (-0.5, -0.2)**	**-0.2 (-0.4, -0.1)**	**.010 [0.47]**	**-0.2 (-0.4, -0.1)**	-0.1 (-0.3, 0.1)	.208 [0.23]
	Control	1.6 (0.1)	-0.1 (-0.2, 0.0)			**-0.1 (-0.3, -0.0)**		
Screen time (average mins/day)								
Children ^e^ (mother proxy)	Intervention	87.5 (6.5)	**-15.9 (-26.8, -5.1)**	-0.1 (-15.5, 15.3)	.989 [0.20]	-6.4 (-17.1, 4.4)	3.9 (-11.4, 19.3)	.614 [0.09]
	Control	100.6 (6.5)	**-15.8 (-26.8, -4.9)**			-10.3 (-21.3, 0.7)		
Fathers ^e^	Intervention	124.1 (6.7)	**-25.2 (-38.1, -12.4)**	**-**16.6 (-34.8, 1.5)	.073 [0.32]	-12.9 (-25.9, 0.3)	-6.3 (-25.1, 12.5)	.509 [0.12]
	Control	108.0 (6.6)	-8.6 (-21.5, 4.3)			-6.6 (-19.9, 6.9)		
Weight status								
Children (BMI-z)	Intervention	0.41 (0.11)	-0.01 (-0.09, 0.09)	-0.01 (-0.14, 0.12)	.892 [0.03]	+ 0.02 (-0.08, 0.11)	-0.11 (-0.24, 0.18)	.092 [0.30]
	Control	0.24 (0.11)	+ 0.00 (-0.09, 0.09)			** + 0.13 (0.03, 0.22)**		
Fathers (BMI)	Intervention	28.0 (0.6)	**-0.3 (-0.5, -0.2)**	-0.2 (-0.4, 0.0)	.061 [0.35]	+ 0.0 (-0.2, 0.3)	-0.1 (-0.4, 0.2)	.548 [0.10]
	Control	28.1 (0.6)	-0.1 (-0.3, 0.0)			+ 0.1 (-0.1, 0.4)		
Fat mass %								
Children ^d^	Intervention	17.8 (0.9)	-0.3 (-2.0, 1.4)	+ 0.5 (-1.9, 2.8)	.695 [0.07]	-1.0 (-2.7, 0.6)	-1.4 (-3.7, 0.9)	.232 [0.22]
	Control	17.5 (0.8)	-0.8 (-2.4, 0.9)			+ 0.4 (-1.3, 2.0)		
Fathers	Intervention	22.3 (1.1)	**-**0.8 (-1.8, 0.2)	-1.0 (-2.4, 0.5)	.184 [0.24]	** + 0.8 (0.2, 1.4)**	-0.2 (-1.0, 0.7)	.687 [0.07]
	Control	23.2 (1.06)	+ 0.2 (-0.8, 1.2)			+ 1.0 (0.4, 1.6)		

Bold denotes a significant difference. *BMI* body mass index, *MVPA* moderate-to-vigorous physical activity, *CPM* counts per minute, *TGMD* Test of Gross Motor Development, *FMS* fundamental movement skills, *Co-PA* co-physical activity. ^a^10 week value minus baseline; ^b^Within-group difference (intervention) minus within-group difference (control); ^c^9 month value minus baseline; ^d^Adjusted for child’s age; ^e^Truncated to account for outliers [58] (> 3.29 SD truncated to next highest value plus 1) ^f^Adjusted for fathers’ age; ^g^Adjusted for SES; ^h^Adjusted for child’s sex. ^i^Adjusted to include additional activity completed without wearing pedometer (e.g., swimming)

^j^Minimum wear-time of 3 days, 7 h/day. ^k^Minimum wear-time of 4 days, 10 h/day

### Secondary outcomes

There were significant intervention effects for fathers’ physical activity levels at 10-weeks (post –intervention), with an increase of 850 steps/day, compared with -177 steps/day in the control group (difference between groups = 1027 steps/day, 95% CI: 157 to 1897, *d* = 0.4). Outcomes for adjusted pedometer step counts (step counts increased to include equivalent steps for documented activity completed without wearing the pedometer e.g., swimming) were consistent with those of unadjusted steps for fathers and children. Significant and sustained intervention effects (all *p* < 0.05) were also identified for the physical activity role modelling (10-weeks: *d* = 0.58, 9-months: *d* = 0.54) and fathers’ screen time parenting practices for: screens as a reward (10-weeks: *d* = 0.49, 9-months: *d* = 0.46).

A large group-by-time effect was detected for children’s object control FMS competence at post-intervention (difference = 4.5 points, 95% CI: 2.5 to 6.5, *d* = 0.8), which was maintained at 9 months (difference = 2.7 points, 95% CI: 0.6 to 4.8, *d* = 0.5). There were no significant group-by-time effects at any time point for children or fathers’ LPA (accelerometer sub-sample), MVPA (accelerometer sub-sample) weight-related outcomes or screen-time, fathers’ self-reported MVPA and father-child co-physical activity. Findings were consistent with those in the completers and per-protocol analyses (Supplementary Tables 2 and 3 in Additional File 1).

### Process evaluation

On average, attendance across the eight sessions for the fathers and children was 86%, while average attendance for the two fathers-only workshops was 96%. Detailed process scores are provided in Table 4. Briefly, fathers considered the timing and structure of the program to be appropriate and overall quality of the program, resources and facilitators to be high. On a scale of 1 (poor) to 5 (excellent), fathers reported a mean (SD) overall program satisfaction score of 4.8 (0.4). Fathers’ mean (SD) satisfaction with the facilitators was 4.9 (0.3).

**Table 4:** Process findings as reported by Fathers (*n* = 55)

Construct	Questions asked^a^	Mean (SD)
Program structure and timing	The timing of the program (Saturday morning) was convenient	4.5 (0.7)
	I felt there was value in having ‘family week’ where mothers/partners and siblings were invited	4.7 (0.7)
Quality of facilitators	Were approachable and warm	4.9 (0.3)
	Were a credible source of information	4.8 (0.4)
	Had a high level of knowledge	4.8 (0.4)
	Had good communication styles (clear, engaging)	4.8 (0.5)
	Were enthusiastic and motivating	4.9 (0.3)
	Displayed good rapport with youngsters	4.9 (0.3)
	Motivated me to apply the knowledge and principles presented in the program	4.7 (0.5)
	Overall rating of facilitators^b^	4.9 (0.3)
Quality of program	The practical activities were appropriate for myself and my youngster	4.6 (0.6)
	The practical activities were appropriate for my fitness levels	4.5 (0.6)
	The information presented at the Dad’s only workshops were relevant to my life	4.3 (0.7)
	The Dad’s only workshops were a worthwhile commitment	4.4 (0.6)
	The Dad’s only workshops added value to the rest of the program	4.4 (0.6)
Impact of program on behaviour	My youngster improved their sport skills as a result of participating in the HYHD program	4.4 (0.7)
Resources (home-based Activity Handbook)	The Weekly home tasks checklist was easy to complete	4.0 (0.6)
	The Weekly home tasks checklist helped me stay on track	4.0 (0.8)
	The activities in the Weekly Home Challenges were easy to complete	4.2 (0.6)
	The activities in the Weekly Sport Skills Practice were easy to complete	3.7 (0.9)
	The Weekly animal character stickers motivated my youngster to complete the weekly home tasks	4.3 (0.9)
	The ‘bonus stickers’ were an additional motivator and encouraged my youngster and I to do additional activities	3.9 (1.0)
	The 'bonus stickers' were an additional motivator and encouraged my youngster and I to wear the pedometer once a week	3.8 (1.0)
Satisfaction	The (dads and youngster) sessions were enjoyable	4.7 (0.4)
	My youngster enjoyed participating in the sessions	4.4 (0.7)
	I would recommend the program to my friends	4.5 (0.4)
	The Dad’s only workshops were enjoyable	4.2 (0.7)
	My youngster enjoyed completing the Weekly Home Challenges	4.4 (0.6)
	I enjoyed completing the Weekly Home Challenges with my youngster	4.4 (0.7)
	My youngster enjoyed completing the Weekly Sport Skills Practice	4.0 (0.9)
	I enjoyed completing the Weekly Sport Skills Practice with my youngster	4.2 (0.7)
	My youngster enjoyed collecting the Weekly animal character stickers	4.8 (0.5)
	Overall program satisfaction^b^	4.8 (0.8)

^a^1 = strongly disagree; 2 = disagree; 3 = neutral; 4 = agree; 5 = strongly agree

^b^ 1 = poor; 2 = fair; 3 = average; 4 = good; 5 = excellent

Detailed fidelity findings are presented in Supplementary Table 4 (Additional File 1). Briefly, on a scale of 1 (strongly disagree) to 5 (strongly agree), facilitators believed there was sufficient time to deliver all content in the dads only workshops (mean 5, SD 0.0) and that fathers were highly engaged in the workshop (mean 5, SD 0.0). On average across the practical sessions involving fathers and children, facilitators delivered 95% of all the required rough and tumble activities, 93% of all the fundamental movement skills activities and 90% of all the fitness activities.

## Discussion

To our knowledge, HYHD is the first lifestyle program internationally that targets fathers and preschool aged children and only one of a few lifestyle programs targeting fathers [34–36]. Compared with the control group, HYHD increased the children’s average daily step count at the primary endpoint (10-weeks) by an additional 1417 steps. This impact was sustained at 9 months with a between-group difference of 1480 steps per day. We also identified significant intervention effects for numerous secondary outcomes including fathers’ physical activity levels, children’s FMS proficiency, and several parenting constructs. There were no significant differences observed between groups at any time-point for fathers’ self-reported MVPA or fathers’ and children’s accelerometer based LPA or MVPA, co-physical activity, screen time and adiposity measures. Process evaluation data revealed very high levels of satisfaction, attendance and retention and the program was delivered as intended with high fidelity findings reported by facilitators.

The children’s physical activity results are promising given the paucity of successful physical activity interventions targeting preschool-aged children [15]. The sustained increase of approximately 1500 steps per day in intervention children compared with control at 9 months is particularly encouraging, especially as our sample had low baseline values of physical activity. One study developed a regression equation which estimated 13,874 daily steps in preschool-aged children to be comparable with the accumulation of 60 min of MVPA [61]. As such, our baseline value of 8837 steps in the HYHD sample falls substantially below this estimation. Our moderate strength effect size of *d* = 0.6 at 9 months appears to be greater when compared to the standardised mean effect size from comparable meta-analyses. Specifically, a meta-analysis of three physical activity interventions in centre-based childcare settings (children aged 0–6 years) demonstrated a standardised mean difference of 0.07 for interventions greater than 6-months and with objective outcome measures (pedometer steps or accelerometer determined MVPA) [62]. Another meta-analysis of 15 physical activity interventions for preschoolers had an effect size of *g* = 0.44 for general physical activity levels, assessed with objective and self-report measures (accelerometers, heart rate monitors, pedometers, direct observation, and parent report). However, effect sizes were much smaller in this meta-analysis when results were stratified by studies > 12 weeks (Hedges *g* = 0.18) and in home based settings (Hedges *g* = 0.28) [63]. A possible explanation for the positive effects in HYHD is due to the program’s adherence to the five key recommendations as reported from a review of family-based physical activity programs [64]. These included: 1) including children as agents of change, 2) ensuring sociocultural tailoring of program, 3) providing education to increase knowledge, 4) targeting social and psychological outcomes and 5) combining goal setting and reinforcement techniques [64]. Furthermore, by engaging fathers, the HYHD program capitalised on the father-child ‘activation relationship’ which is primarily developed through physical play [65]. Ultimately, this relationship can heighten the bond between fathers and children, leading to a range of holistic benefits to children [25, 65].

Our substantive and sustained improvements in FMS proficiency may have contributed to the children's physical activity intervention effects. Mastery in FMS is associated with higher levels of physical activity participation in preschool children [66]. Our FMS improvement in object control score (mean difference of 4.5 points), which was largely maintained at 9 months (mean difference of 2.7 points) is greater than has been observed in other programs for preschool children, with a recent meta-analysis detecting a standardised mean difference of 1.06 (95%CI: 0.46, 1.66) on object control skills [67]. This is likely due to the one-to-one support from fathers at the HYHD sessions and at home, which maximises contact time to learn these skills. Furthermore, as fathers often provide a better model of sports skill performance [21], children can learn and mirror the correct technique to optimise proficiency of these skills.

For the paternal physical activity outcomes, we identified significant intervention effects at 10-weeks (+ 1027 steps/day) which were no longer significant at 9 months (+ 633 steps/day 95%CI: -254, 1520). The slight increase in steps among the control group (+ 104 steps/day) at 9 months may have attenuated the overall effect. Despite this, additional strategies may be required for fathers to maintain these effects long term. Overall, our positive findings are comparable with those observed in the two previous interventions that targeted fathers [34–36]. Additionally, the effect size for steps in our study (*d* = 0.5) and other trials targeting fathers [34–36] are larger than reported in most physical activity interventions targeting men in general [68, 69]. This could be attributed to the targeted reciprocal reinforcement (e.g., father and children encourage each other be active), valued outcomes (e.g., enhancing the father-child relationship in addition to health improvements), and co-physical activity among the father and child programs.

Our findings suggest that children may have increased overall steps rather than higher intensity physical activity due to no intervention effects observed at any time-point for accelerometer-determined MVPA in a sub-sample of children (*n* = 43). This aligns with the focus of the program which was to improve overall physical activity rather than promoting more vigorous physical activity. Despite accelerometers being gold standard, budgetary constraints warranted the use of pedometers as the primary outcome and only measure LPA and MVPA using accelerometers in a small sub-sample. As such we were not powered to detect changes in accelerometer based LPA or MVPA.

The acceptability of HYHD was established through very high levels of attendance (86% for fathers and children), retention (78% at 9 months) and satisfaction (mean overall program satisfaction score of 4.8 out of 5). Our high attendance and retention rates are similar to other programs that targeted the father-child in the community (“Healthy Dads, Healthy Kids” Community randomized control trial: attendance = 71%, retention = 81% [34]) and parent-preschool-aged child (“MEND 2–4”: attendance = 82%, retention = 86% [70]). Our high attendance and retention rates may be due to high participant satisfaction with overall quality of the program, resources and facilitators. Overall, our high acceptability shows that fathers and children are willing to engage with behaviour change interventions that are specifically targeted to suit their unique preferences and values. This provides further evidence of the potential for socio-culturally targeted interventions to engage fathers in health research and improve family health outcomes [71].

Strengths of our study include: successful targeting and recruitment of fathers and their preschool-aged children, a randomised controlled design, intention-to-treat analyses, objective physical activity data, follow-up assessments 9 months after baseline and high retention. Limitations include potential reporting bias from self-report measures and skewed participation towards more active and socioeconomically advantaged fathers and children. Also, a wait-list control group was used rather than an attention-placebo control group. Therefore, the study was unable to determine whether the HYHD program increased children’s daily steps above what may have been observed by increasing father-child interactions in other contexts. However, due to the scarcity of physical activity interventions targeting fathers and their preschool-aged children in the literature, the authors believe the decision to use a wait-list control was justified. In addition, the statistical and ethical complexities associated with designing and implementing attention-placebo controls for behavioural medicine trials [72] provide additional justification for this approach. The physical activity assessment (pedometer steps/day) may have been somewhat affected by reactivity, as intervention participants also used a pedometer as part of the intervention. However, we ensured that any weeks where participants documented their steps for assessment purposes did not overlap with the action intervention period. There is contradicting evidence regarding validity of pedometers with preschool children [38, 39, 61, 73, 74]. However, the feasibility of this measure has been established in this age group [32] and correlation studies have shown moderate associations with direct observations and accelerometry [61, 75]. Specifically, previous research has established convergent validity of the Yamax SW-200 pedometer (*r* = 0.73) when compared MTI 7164 ActiGraph accelerometer [13].

## Conclusion

This was the first physical activity program internationally targeting fathers to become healthy lifestyle role models for their preschool-aged children, and vice versa. The sustained improvements in physical activity among the children supported the study hypotheses. In addition, improvements in secondary outcomes further support engaging fathers to improve family health outcomes. Further research is needed to confirm the effectiveness and scalability of the program when delivered in community settings by trained facilitators to a more diverse range of families.

## Supplementary Information

**Additional file 1: Supplementary Table 1.** Description of intervention components in the ‘Healthy Youngsters, Healthy Dads’ program. **Supplementary Table 2.** Changes in primary and secondary outcomes for study participants (per-protocol). **Supplementary Table 3**. Changes in primary and secondary outcomes for study participants (completers). **Supplementary Table 4. **Facilitator reflections and fidelity findings.

## Data Availability

The de-identified data are available from PJM upon reasonable request.

## Abbreviations

HYHD: Healthy Youngsters, Healthy Dads,
FMS: Fundamental Movement Skills,
MVPA: Moderate-to-Vigorous Physical Activity
LPA: Light Physical Activity
MPA: Moderate Physical Activity
VPA: Vigorous Physical Activity
BMI: Body Mass Index
RCT: Randomised Controlled Trial
ITT: Intention to Treat
CI: Confidence Intervals
CONSORT: Consolidated Standards of Reporting Trials
CPM: Counts per minute
TGMD: Test of Gross Motor Development
LMS: Least Mean Square
SEIFA: Socio-Economic Indexes for Areas
