# Supplementary material for: Impact of the ‘Healthy Youngsters, Healthy Dads’ program on physical activity and other health behaviours: a randomised controlled trial involving fathers and their preschool-aged children
Source: BMC Public Health. 2022 Jun 10;22:1166. doi: 10.1186/s12889-022-13424-1 (PMC9188227; doi:10.1186/s12889-022-13424-1)
Supplement: Supplementary file 1 — Additional file 1: Supplementary Table 1. Description of intervention components in the ‘Healthy Youngsters, Healthy Dads’ program. Supplementary Table 2. Changes in primary and secondary outcomes for study participants (per-protocol). Supplementary Table 3. Changes in primary and secondary outcomes for study participants (completers). Supplementary Table 4. Facilitator reflections and fidelity findings. [file 12889_2022_13424_MOESM1_ESM.docx]

Additional File 1 – Supplementary Tables

**Impact of the ‘Healthy Youngsters, Healthy Dads’ program on physical activity and other health behaviours: A randomised controlled trial involving fathers and their pre-school aged children**

Philip J. Morgan*, Jacqueline A. Grounds, Lee M Ashton, Clare E. Collins, Alyce T. Barnes, Emma R. Pollock, Stevie-Lee Kennedy, Anna T. Rayward, Kristen L. Saunders, Ryan J Drew, Myles D. Young

***** Correspondence: Philip.morgan@newcastle.edu.au; Tel.: +61-2-49-217265

**Contents**

| **Supporting information item** | **Page** |
| --- | --- |
| **Supplementary Table 1.** Description of intervention components in the ‘Healthy Youngsters, Healthy Dads’ program | 2 |
| **Supplementary Table 2.** Changes in primary and secondary outcomes for study participants (per-protocol) | 4 |
| **Supplementary Table 3**. Changes in primary and secondary outcomes for study participants (completers) | 6 |
| **Supplementary Table 4:** Facilitator reflections and fidelity findings | 8 |

| **Supplementary Table 1.** Description of intervention components in the ‘Healthy Youngsters, Healthy Dads’ program | | | | |
| --- | --- | --- | --- | --- |
| **Intervention component** | **Overview** | **Detail** | **Behaviour change techniques** | **Targeted theoretical mediators** |
| ‘Fathers-only’ workshop | - 2 x 2hours delivered by a trained facilitator with physical education qualifications. - Held on the Thursday evenings before and after the first father-and-child session. | Topics covered:   - Optimising health in the early years - The unique and powerful influence of fathers - Positive parenting strategies - SMART goal setting - Physical activity parenting - Fundamental movement skills - Healthy eating for families - Screen-time parenting | • Social support (practical, emotional)  • Increase positive emotions  • Instructions on how to perform the behaviour  • Information about consequences (health, social and environmental, emotional)  • Demonstration of the behaviour  • Graded tasks  • Credible source  • Identification of self as role model  • Framing/reframing  • Verbal persuasion about capability | - Social support/ relatedness (SCT/SDT) - Autonomy (SDT) - Self-efficacy/ perceived competence (SCT/SDT) - Outcome expectations (SCT) - Goals (SCT) |
| Weekly group sessions for fathers and children | - 8 x weekly 75-minute sessions (20-minute *Education session* plus 55-minute *Practical session).* - Mothers/partners and siblings invited to attend week 5 session. - Four facilitators were trained to deliver the program, and each session was delivered by two facilitators. All facilitators had education qualifications. - Participants were offered one of three Saturday morning timeslots, delivered by two facilitators. Some facilitators delivered more than one session each week. | *Education session:*   - Icebreakers and overview of weekly focus which alternated between physical activity (e.g. rough and tumble play, sport skills) and healthy eating (e.g. vegetables, fruit). - Each week introduced a new animal character and a slogan.   *Practical session:*   - Designed to increase preschool-aged children’s motivation and skills to engage in physical activity fun and active father-child games. - Each session targeted rough and tumble play (15 min), sport skills (i.e., FMS) (15 min), and aerobic and muscular fitness (15 min). There was a cool-down component (5 mins) at the end of every session and transition time was accounted for (5 mins). - Seven sports skills targeted (2/week): catch, kick, one-handed strike, two-handed strike, bounce, overhand throw and underhand throw. |  |  |
| Home-based program | - Activity Handbook containing a range of engaging activities, challenges and sport skills. - One Yamax SW200 *pedometer* to assist with monitoring step counts. - Sticker chart to earn the animal sticker of the week (e.g. Charlie the Chimpanzee) for one home challenge and bonus stickers for completing more than one activity (e.g. apple, basketball). - One home challenge task was minimum requirement. | Fathers asked to use Activity Handbook each week and record completed home tasks:   - Home challenges based on weekly theme (e.g., sock wrestle) - Sport skill games (e.g., capture the target) - Step count monitoring (using pedometer) - Co-physical activity (≥ 10-minute bouts) - SMART goals relating to physical activity, healthy eating, screen time and parenting - Review SMART goal progress - Dad task (e.g. bring at least one piece of fruit with you to work every day this week.) | - Material incentive   - Instructions on how to perform the behaviour   - Graded tasks   - Prompts/cues   - Increase positive emotions   - Goal setting   - Action planning   - Self-monitoring | - - Goals (SCT)   - Social support/ relatedness (SCT/SDT)   - Autonomy   - Self-efficacy /perceived   competence (SCT/ SDT) |

Abbreviations: SCT = social cognitive theory; SDT = self-determination theory; FMS= fundamental movement skills; SMART = Specific, Measurable, Achievable, Relevant, Timely

| **Supplementary Table 2.** Changes in primary and secondary outcomes for study participants (per-protocol) | | | | | | | | |
| --- | --- | --- | --- | --- | --- | --- | --- | --- |
|  |  | **Baseline** | **10 weeks change from baseline**  **(Mean, 95% CI)** | | | **9 months change from baseline**  **(Mean, 95% CI)** | | |
| **Outcome** | **Group** | **Mean (SE)** | **Within group^a^** | **Mean difference between groups^b^** | ***p*-value [Cohen’s *d*]** | **Within group^c^** | **Mean difference between groups^b^** | ***p*-value [Cohen’s *d*]** |
| Primary Outcome |  |  |  |  |  |  |  |  |
| Steps/day |  |  |  |  |  |  |  |  |
| Children  (n=89)^d, e^ | Intervention | 7960 (453) | **+2145 (1234, 3055)** | **+1661 (526, 2795)** | **.004  [0.61]** | **+1647 (691, 2604)** | +1151 (-23, 2325) | .055  [0.41] |
|  | Control | 9558 (327) | +484 (-193, 1160) |  |  | +497 (-184, 1177) |  |  |
| Secondary Outcomes |  |  |  |  |  |  |  |  |
| Steps/day |  |  |  |  |  |  |  |  |
| Fathers (n=90)^d^ | Intervention | 8146 (505) | **+1225 (419, 2031)** | **+1400 (392, 2408)** | **.007  [0.58]** | **+903 (54, 1751)** | +797 (-247, 1842) | .134  [0.32] |
|  | Control | 8160 (366) | -175 (-781, 430) |  |  | +106 (-504, 715) |  |  |
| Adjusted steps/day^i^ | | | | | | | | |
| Children ^d,e,f^ | Intervention | 10,371 (575) | +731 (-504, 1966) | **1799 (261, 3337)** | **0.022 [0.49]** | +1139 (-157, 2435) | **1909 (319, 3500)** | **0.019 [0.50]** |
|  | Control | 12,050 (415) | **-1068 (-1985, -151)** |  |  | -770 (-1693, 153) |  |  |
| Fathers ^d^ | Intervention | 9544 (634) | +908 (-247, 2064) | **+1731 (287, 3175)** | **0.019 [0.50]** | +638 (-576, 1853) | 1311 (-184, 2807) | 0.085 [0.37] |
|  | Control | 9824 (459) | -822 (-1689, 44) |  |  | -673 (-1546, 200) |  |  |
| LPA (accelerometer sub-sample) (mins/d) | | | | | | | | |
| Children (n=32)^≠^ | Intervention | 246 (10) | -2 (-24, 20) | 0 (-27, 26) | 0.971  [0.01] | +5 (-18, 28) | -4 (-31, 24) | 0.783  [-0.10] |
|  | Control | 246 (7) | -2 (-17, 14) |  |  | +9 (-6, 24) |  |  |
| Fathers (n=34)^*^ | Intervention | 169 (14) | **-53 (-86, -20)** | -9 (-49, 31) | 0.654  [-0.16] | -15 (-50, 21) | -10 (-52, 33) | 0.661  [-0.15] |
|  | Control | 186 (11) | **-44 (-67, -20)** |  |  | -5 (-29, 18) |  |  |
| MVPA (accelerometer sub-sample) (mins/d) | | | | | | | | |
| Children (n=32)^≠^ | Intervention | 98 (9) | +12 (-8, 31) | +2 (-21, 26) | 0.841  [0.07] | **+24 (4, 44)** | +4 (-20, 29) | 0.726  [0.12] |
|  | Control | 108 (6) | +9 (-4, 22) |  |  | **+20 (6, 33)** |  |  |
| Fathers (n=34)^*^ | Intervention | 64 (15) | **+51 (1, 101)** | +10 (-51, 72) | 0.740  [0.12] | -4.2 (-32, 24) | +1 (-33, 35) | 0.956  [0.02] |
|  | Control | 90 (11) | **+41 (5, 76)** |  |  | -5 (-24, 14) |  |  |
| Fathers’ self-reported MVPA (mins/wk) | | | | | | | | |
| (n=95)^d^ | Intervention | 126 (36) | +59 (-4, 121) | +59 (-18, 136) | .128  [0.32] | +40 (-33, 112) | +63 (-27, 152) | .165  [0.29] |
|  | Control | 174 (25) | +0.7 (-46, 44) |  |  | -23 (-76, 29) |  |  |
| Children’s FMS competence (TGMD) | | | | | | | | |
| Object control  score (n=94)^e, f^ | Intervention | 9.4 (0.9) | **+4.7 (2.8, 6.5)** | **+4.4 (2.1, 6.7)** | **.000  [0.77]** | **+7.1 (5.3, 9.0)** | +2.0 (-0.4, 4.4) | .095 [0.35] |
|  | Control | 10.6 (0.6) | +0.3 (-1.1, 1.6) |  |  | **+5.1 (3.7, 6.6)** |  |  |
| Co-physical activity (days/wk) | | | | | | | | |
| 1-on-1 (n=95) | Intervention | 1.8 (0.3) | **+1.0 (0.4, 1.6)** | +0.5 (-0.2, 1.2) | .182  [0.28] | +0.1 (-0.5, 0.7) | -0.1 (-0.8, 0.6) | .784 [0.06] |
|  | Control | 1.3 (0.2) | **+0.5 (0.0, 0.9)** |  |  | +0.2 (-0.2, 0.7) |  |  |
| Family (other children or family) (n=95)^f^ | Intervention | 2.7 (0.3) | +0.5 (-0.0, 1.1) | +0.5 (-0.2, 1.2) | .179  [0.28] | +0.1 (-0.5, 0.7) | +0.1 (-0.6, 0.8) | .792  [0.05] |
|  | Control | 2.3 (0.2) | +0.0 (-0.4, 0.5) |  |  | -0.0 (-0.5, 0.4) |  |  |
| Fathers’ role modelling | | | | | | | | |
| Physical Activity  (n=95) | Intervention | 2.6 (0.1) | **+0.5 (0.3, 0.7)** | **+0.4 (0.2, 0.9)** | **.000  [0.81]** | **+0.3 (0.2, 0.5)** | **+0.3 (0.1, 0.5)** | **.004  [0.59]** |
|  | Control | 2.7 (0.1) | +0.1 (-0.0, 0.2) |  |  | +0.0 (-0.1, 0.2) |  |  |
| Screen time parenting practices | | | | | | | | |
| Fathers’ screen as  reward (n=95)^d^ | Intervention | 2.3 (0.1) | **-0.6 (-0.9, -0.4)** | **-0.4 (-0.7, -0.1)** | **.008  [0.55]** | **-0.6 (-0.8, -0.4)** | **-0.3 (-0.6, -0.1)** | **.019  [0.48]** |
|  | Control | 2.3 (0.1) | **-0.3 (-0.4, -0.1)** |  |  | **-0.3 (-0.4, -0.1)** |  |  |
| Fathers’ screens  other than TV  (n =95)^f, g^ | Intervention | 1.6 (0.1) | **-0.4 (-0.5, -0.2)** | **-0.2 (-0.4, -0.1)** | **.012  [0.53]** | **-0.3 (-0.4, -0.1)** | -0.1 (-0.3, 0.1) | .247  [0.24] |
|  | Control | 1.6 (0.1) | **-0.1 (-0.2, -0.0)** |  |  | **-0.1 (-0.3, -0.0)** |  |  |
| Screen time (average mins/day) | | | | | | | | |
| Children (n =91)  (mother proxy)^d^ | Intervention | 83.9 (9.7) | -12.5 (-27.4, 2.4) | +3.3 (-15.5, 22.2) | .727  [0.07] | -5.4 (-20.4, 9.7) | +4.9 (-14.1, 24.0) | .608 [0.11] |
|  | Control | 100.6 (7.0) | **-15.9 (-27.5, -4.2)** |  |  | -10.3 (-21.9, 1.3) |  |  |
| Fathers (n=95) ^d^ | Intervention | 115.0 (9.2) | **-20.5 (-37.4, -3.6)** | **-**11.9 (-32.9, 9.2) | .267 [0.23] | -12.6 (-29.5, 4.3) | -6.0 (-27.4, 15.3) | .577 [0.11] |
|  | Control | 108.0 (6.4) | -8.6 (-21.5, 4.0) |  |  | -6.5 (-19.6, 6.6) |  |  |
| Weight status |  |  |  |  |  |  |  |  |
| Children (BMI-z)  (n=94) | Intervention | 0.61 (0.16) | -0.02 (-0.14, 0.10) | -0.02 (-0.17, 0.13) | .770  [0.06] | +0.03 (-0.15, 0.09) | **-0.16 (-0.31, -0.01)** | **.041  [0.43]** |
|  | Control | 0.24 (0.11) | +0.00 (-0.09, 0.09) |  |  | **+0.13 (0.03, 0.22)** |  |  |
| Fathers (BMI)  (n=95) | Intervention | 28.0 (0.9) | **-0.4 (-0.6, -0.2)** | -0.2 (-0.5, 0.0) | .067  [0.38] | +0.1 (-0.3, 0.4) | -0.1 (-0.5, 0.3) | .708  [0.08] |
|  | Control | 28.2 (0.6) | -0.1 (-0.3, 0.0) |  |  | +0.1 (-0.1, 0.4) |  |  |
| Fat mass % | | | | | | | | |
| Children (n=94)^d, h^ | Intervention | 18.6 (1.1) | +0.2 (-2.0, 2.5) | +1.0 (-1.7, 3.7) | .474  [0.15] | -0.7 (-2.6, 1.1) | -1.1 (-3.4, 1.3) | .360 [0.19] |
|  | Control | 17.5 (0.8) | -0.8 (-2.2, 0.8) |  |  | +0.4 (-1.1, 1.8) |  |  |
| Fathers (n =94)^f^ | Intervention | 23.7 (1.5) | **-**1.2 (-2.7, 0.2) | -1.4 (-3.2, 0.4) | .133  [0.31] | +0.6 (-0.1, 1.2) | -0.4 (-1.2, 0.5) | .687  [0.17] |
|  | Control | 23.3 (1.0) | +0.1 (-1.0, 1.2) |  |  | **+0.9 (0.4, 1.4)** |  |  |

Bold denotes a significant difference. MVPA moderate-to-vigorous physical activity. a. 10 week value minus baseline; b. Within-group difference (intervention) minus within-group difference (control); c. 9 month value minus baseline; d. Truncated to account for outliers (1) (>3.29 SD truncated to next highest value plus 1) e. Adjusted for child’s sex; f. Adjusted for SES; g. Adjusted for fathers’ age; h. Adjusted for child’s age. i. Adjusted to include additional activity completed without wearing pedometer (e.g., swimming). ^≠^Minimum wear-time of 3 days, 7hrs/day. *Minimum wear-time of 4 days, 10hrs/day.

| **Supplementary Table 3.** Changes in primary and secondary outcomes for study participants (completers) | | | | | | | | |
| --- | --- | --- | --- | --- | --- | --- | --- | --- |
|  |  | **Baseline** | **10 weeks change from baseline**  **(Mean, 95% CI)** | | | **9 months change from baseline**  **(Mean, 95% CI)** | | |
| **Outcome** | **Group** | **Mean (SE)** | **Within group^a^** | **Mean difference between groups^b^** | ***p*-value [Cohen’s *d*]** | **Within group^c^** | **Mean difference between groups^b^** | ***p*-value [Cohen’s *d*]** |
| Primary Outcome |  |  |  |  |  |  |  |  |
| Steps/day |  |  |  |  |  |  |  |  |
| Children (n=95)^d, e^ | Intervention | 7974 (384) | **+1936 (1194, 2678)** | **+1245 (221, 2270)** | **.017 [0.49]** | **+2092 (1348, 2835)** | **+1461 (432, 2491)** | **.006 [0.57]** |
|  | Control | 9285 (366) | +691 (-16, 1397) |  |  | +630 (-82, 1342) |  |  |
| Secondary Outcomes |  |  |  |  |  |  |  |  |
| Steps/day |  |  |  |  |  |  |  |  |
| Father (n=95)^d^ | Intervention | 8317 (420) | **+805 (174, 1436)** | **+1002 (118, 1886)** | **.027 [0.46]** | **+773 (142, 1404)** | +675 (-209, 1559) | .133 [0.31] |
|  | Control | 8500 (414) | -197 (-816, 423) |  |  | +98 (-521, 717) |  |  |
| Adjusted steps/day^i^ | | | | | | | | |
| Children ^d,e^ | Intervention | 10,511 (546) | +716 (-220, 1651) | **+1628 (339, 2918)** | **0.014 [0.51]** | **+1423 (249, 2596)** | **+2147 (523, 3771)** | **0.010 [0.54]** |
|  | Control | 11,921 (521) | **-913 (-1800, -25)** |  |  | -724 (-1847, 398) |  |  |
| Fathers ^d^ | Intervention | 10,427 (524) | +13.6 (-927, 954) | +965 (-354, 2284) | 0.150 [0.30] | +266 (-675, 1206) | +1053 (-266, 2371) | 0.117 [0.32] |
|  | Control | 10,219 (516) | **-951 (-1875, -28)** |  |  | -787 (-1711, 137) |  |  |
| LPA (accelerometer sub-sample) (mins/d) | | | | | | | | |
| Children (n =34) ^≠^ | Intervention | 256 (8) | -7 (-24, 11) | -8 (-31, 15) | 0.495  [-0.24] | 0 (-18, 17) | -9 (-33, 14) | 0.435  [-0.27] |
|  | Control | 243 (7) | +1 (-14, 17) |  |  | +9 (-7, 25) |  |  |
| Fathers (n=32)* | Intervention | 182 (13) | **-53 (-81, -26)** | -2 (-38, 34) | 0.902  [-0.04] | -11 (-39, 16) | -2 (-38, 34) | 0.901 [-0.04] |
|  | Control | 190 (11) | **-51 (-74, -28)** |  |  | -9 (-32, 14) |  |  |
| MVPA (accelerometer sub-sample) (mins/d) | | | | | | | | |
| Children (n =34) ^≠^ | Intervention | 104 (7) | +7 (-8, 21) | -6 (-25, 13) | 0.547  [-0.20] | **+23 (8, 37)** | +4 (-15, 23) | 0.682  [0.14] |
|  | Control | 109 (6) | +12 (-0, 25) |  |  | **+19 (6, 31)** |  |  |
| Fathers (n=32)* | Intervention | 76 (15) | +45 (-1, 90) | +3 (-55, 62) | 0.905  [0.04] | -3 (-24, 19) | +4 (-24, 32) | 0.771  [0.10] |
|  | Control | 94 (12) | **+42 (4, 79)** |  |  | -7 (-25, 11) |  |  |
| Fathers’ self-reported MVPA (mins/wk) | | | | | | | | |
| (n=99)^d^ | Intervention | 145 (24) | **+61 (18, 104)** | +31 (-31, 93) | .326 [0.20] | +30 (-21, 80) | +22 (-51, 95) | .556 [0.12] |
|  | Control | 143 (25) | +30 (-15, 75) |  |  | +8 (-45, 61) |  |  |
| Children’s FMS competence (TGMD) | | | | | | | | |
| Object control  score (n=97)^e, f, g^ | Intervention | 9.0 (0.7) | **+4.9 (3.4, 6.5)** | **+4.5 (2.3, 6.8)** | **.000  [0.82]** | **+8.0 (6.4, 9.5)** | **+3.0 (0.7, 5.2)** | **.009  [0.54]** |
|  | Control | 10.8 (0.7) | +0.4 (-1.2, 2.0) |  |  | **+5.0 (3.4, 6.6)** |  |  |
| Co-physical activity (days/wk) | | | | | | | | |
| 1-on-1 (n=99) | Intervention | 1.6 (0.2) | **+0.9 (0.4, 1.4)** | +0.4 (-0.3, 1.1) | .264 [0.23] | +0.4 (-0.1, 0.9) | +0.2 (-0.5, 0.9) | .565 [0.12] |
|  | Control | 1.4 (0.2) | +0.5 (-0.1, 1.0) |  |  | +0.2 (-0.4, 0.7) |  |  |
| Family (other children or family) (n=99) | Intervention | 2.7 (0.2) | +0.3 (-0.2, 0.7) | +0.1 (-0.6, 0.8) | .868 [0.03] | -0.0 (-0.5, 0.4) | -0.1 (-0.8, 0.6) | .818 [0.05] |
|  | Control | 2.3 (0.3) | +0.2 (-0.3, 0.7) |  |  | -0.0 (-0.5, 0.5) |  |  |
| Fathers’ role modelling | | | | | | | | |
| Physical Activity  (n=99) | Intervention | 2.7 (0.1) | **+0.4 (0.3, 0.5)** | **+0.3 (0.1, 0.5)** | **.002  [0.65]** | **+0.3 (0.2, 0.5)** | **+0.3 (0.1, 0.5)** | **.004  [0.59]** |
|  | Control | 2.7 (0.1) | +0.1 (-0.0, 0.2) |  |  | +0.0 (-0.1, 0.2) |  |  |
| Screen time parenting practices | | | | | | | | |
| Fathers’ screen as  reward (n=99)^d^ | Intervention | 2.4 (0.1) | **-0.6 (-0.8, -0.4)** | **-0.3 (-0.5, -0.0)** | **.025  [0.45]** | **-0.6 (-0.8, -0.4)** | **-0.3 (-0.5, -0.0)** | **.031  [0.44]** |
|  | Control | 2.4 (0.1) | **-0.3 (-0.5, -0.1)** |  |  | **-0.3 (-0.5, -0.1)** |  |  |
| Fathers’ screens  other than TV  (n =99)^f, h^ | Intervention | 1.6 (0.7) | **-0.4 (-0.5, -0.3)** | **-0.3 (-0.5, -0.1)** | **.002  [0.62]** | **-0.3 (-0.4, -0.1)** | -0.2 (-0.3, 0.0) | .072  [0.36] |
|  | Control | 1.6 (0.1) | -0.1 (-0.2, 0.0) |  |  | -0.1 (-0.2, 0.0) |  |  |
| Screen time (average mins/day) | | | | | | | | |
| Children (n =85)^d^  (mother proxy) | Intervention | 87.5 (7.7) | **-15.3 (-27.0, -3.5)** | +8.0 (-8.9, 25.0) | .350  [0.20] | -6.9 (-18.6, 4.9) | +9.8 (-7.2, 26.7) | .257 [0.25] |
|  | Control | 114.0 (8.0) | **-23.3 (-35.5, -11.1)** |  |  | **-16.6 (-28.8, -4.5)** |  |  |
| Fathers (n=99)^d^ | Intervention | 125.1 (7.4) | **-26.0 (-39.3, -12.7)** | **-**18.1 (-37.4, 1.2) | .065 [0.37] | **-13.4 (-26.7, -0.1)** | -4.8 (-24.1, 14.5) | .625 [0.10] |
|  | Control | 113.7 (7.8) | -7.9 (-21.8, 6.1) |  |  | -8.6 (-22.6, 5.4) |  |  |
| Weight status |  |  |  |  |  |  |  |  |
| Children (BMI-z)  (n=109) | Intervention | 0.37 (0.12) | -0.00 (-0.09, 0.09) | -0.00 (-0.13, 0.12) | .973 [0.01] | +0.02 (-0.08, 0.11) | -0.10 (-0.23, 0.03) | .131 [0.29] |
|  | Control | 0.24 (0.12) | +0.00 (-0.09, 0.09) |  |  | +0.12 (0.03, 0.21) |  |  |
| Fathers (BMI)  (n=99) | Intervention | 27.6 (0.7) | **-0.3 (-0.5, -0.1)** | -0.2 (-0.5, 0.1) | .115 [0.32] | +0.0 (-0.2, 0.3) | -0.1 (-0.4, 0.3) | .765 [0.06] |
|  | Control | 28.2 (0.7) | -0.1 (-0.4, 0.1) |  |  | +0.1 (-0.1, 0.3) |  |  |
| Fat mass % | | | | | | | | |
| Children (n=85)^f, g^ | Intervention | 16.4 (0.9) | +0.2 (-1.7, 2.0) | +0.9 (-1.7, 3.4) | .500 [0.15] | -1.0 (-2.7, 0.8) | -1.4 (-3.8, 1.1) | .274 [0.24] |
|  | Control | 17.6 (0.8) | -0.7 (-2.4, 1.0) |  |  | +0.4 (-1.3, 2.1) |  |  |
| Fathers (n =97) | Intervention | 22.2 (1.2) | **-**0.7 (-1.5, 0.0) | -0.4 (-1.5, 0.6) | .446 [0.16] | **+0.8 (0.4, 1.6)** | -0.1 (-1.2, 1.0) | .880 [0.03] |
|  | Control | 23.5 (1.2) | -0.3 (-1.1, 0.5) |  |  | **+0.9 (0.1, 1.6)** |  |  |

Bold denotes a significant difference. MVPA moderate-to-vigorous physical activity. a. 10 week value minus baseline; b. Within-group difference (intervention) minus within-group difference (control); c. 9 month value minus baseline; d. Truncated to account for outliers (1) (>3.29 SD truncated to next highest value plus 1) e. Adjusted for child’s sex;. f. Adjusted for SES; g. Adjusted for child’s age; h. Adjusted for fathers’ age. i. Adjusted to include additional activity completed without wearing pedometer (e.g., swimming).

^≠^Minimum wear-time of 3 days, 7hrs/day. *Minimum wear-time of 4 days, 10hrs/day.

**Supplementary Table 4:** Facilitator reflections and fidelity findings

| **Session** | **Number of facilitators** | **Number of sessions delivered^≠^** | **Questions asked** | **Mean (SD)* or n (%)** |
| --- | --- | --- | --- | --- |
| Dads workshop 1 | 2 | 2 | The fathers were engaged in the workshop | 5.0 (0.0) |
|  |  |  | There was sufficient time to get through all the content | 5.0 (0.0) |
|  |  |  | There was sufficient time to adequately cover discussion points and answer any questions | 5.0 (0.0) |
|  |  |  | The discussion points/stand up activity slides were well spaced throughout the workshop | 5.0 (0.0) |
| Dads workshop 2 | 2 | 2 | The fathers were engaged in the workshop | 5.0 (0.0) |
|  |  |  | There was sufficient time to get through all the content | 5.0 (0.0) |
|  |  |  | There was sufficient time to adequately cover discussion points and answer any questions | 5.0 (0.0) |
|  |  |  | The discussion points/stand up activity slides were well spaced throughout the workshop | 5.0 (0.0) |
| Session 1 (Dad and Youngster session) | 4 | 6 | The fathers were engaged in the 'Welcome session' | 4.7 (0.5) |
|  |  |  | The youngsters were engaged in the 'Welcome session' | 4.7 (0.8) |
|  |  |  | The dads enjoyed the practical session | 4.8 (0.4) |
|  |  |  | The youngsters enjoyed the practical session | 4.8 (0.4) |
|  |  |  | N (%) of sessions where all required 10 rough and tumble activities were delivered | 5 (83%) |
|  |  |  | N (%) of sessions where all required 5 fundamental movement skill activities were delivered | 6 (100%) |
|  |  |  | N (%) of sessions where all required 4 fitness activities were delivered | 6 (100%) |
| Session 2 (Dad and Youngster session) | 4 | 6 | The fathers were engaged in the 'Welcome session' | 4.5 (0.5) |
|  |  |  | The youngsters were engaged in the 'Welcome session' | 4.7 (0.5) |
|  |  |  | The dads enjoyed the practical session | 4.7 (0.5) |
|  |  |  | The youngsters enjoyed the practical session | 4.7 (0.5) |
|  |  |  | N (%) of sessions where all required 8 rough and tumble activities were delivered | 6 (100%) |
|  |  |  | N (%) of sessions where all required 5 fundamental movement skill activities were delivered | 6 (100%) |
|  |  |  | N (%) of sessions where all required 3 fitness activities were delivered | 4 (67%) |
| Session 3 (Dad and Youngster session) | 3 | 6 | The fathers were engaged in the 'Welcome session' | 4.3 (0.5) |
|  |  |  | The youngsters were engaged in the 'Welcome session' | 4.5 (0.5) |
|  |  |  | The dads enjoyed the practical session | 4.3 (0.5) |
|  |  |  | The youngsters enjoyed the practical session | 4.5 (0.5) |
|  |  |  | N (%) of sessions where all required 7 rough and tumble activities were delivered | 6 (100%) |
|  |  |  | N (%) of sessions where all required 4 fundamental movement skill activities were delivered | 6 (100%) |
|  |  |  | N (%) of sessions where all required 3 fitness activities were delivered | 4 (67%) |
| Session 4 (Dad and Youngster session) | 4 | 6 | The fathers were engaged in the 'Welcome session' | 4.3 (0.8) |
|  |  |  | The youngsters were engaged in the 'Welcome session' | 4.5 (0.8) |
|  |  |  | The dads enjoyed the practical session | 4.8 (0.4) |
|  |  |  | The youngsters enjoyed the practical session | 4.8 (0.4) |
|  |  |  | N (%) of sessions where all required 5 rough and tumble activities were delivered | 6 (100%) |
|  |  |  | N (%) of sessions where all required 4 fundamental movement skill activities were delivered | 6 (100%) |
|  |  |  | N (%) of sessions where all required 3 fitness activities were delivered | 6 (100%) |
| Session 5 (Dad and Youngster session) | 4 | 6 | The fathers were engaged in the 'Welcome session' | 4.2 (0.4) |
|  |  |  | The youngsters were engaged in the 'Welcome session' | 4.3 (0.5) |
|  |  |  | Other family members were engaged in the 'Welcome session' | 4.5 (0.5) |
|  |  |  | The dads enjoyed the practical session | 4.8 (0.4) |
|  |  |  | The youngsters enjoyed the practical session | 4.7 (0.5) |
|  |  |  | Other family members enjoyed the practical session | 4.8 (0.4) |
|  |  |  | N (%) of sessions where all required 11 rough and tumble activities were delivered | 6 (100%) |
|  |  |  | N (%) of sessions where all required 4 fundamental movement skill activities were delivered | 6 (100%) |
|  |  |  | N (%) of sessions where all required 3 fitness activities were delivered | 5 (83%) |
| Session 6 (Dad and Youngster session) | 4 | 6 | The fathers were engaged in the 'Welcome session' | 4.3 (0.5) |
|  |  |  | The youngsters were engaged in the 'Welcome session' | 4.5 (0.5) |
|  |  |  | The dads enjoyed the practical session | 4.8 (0.4) |
|  |  |  | The youngsters enjoyed the practical session | 4.8 (0.4) |
|  |  |  | N (%) of sessions where all required 5 rough and tumble activities were delivered | 6 (100%) |
|  |  |  | N (%) of sessions where all required 5 fundamental movement skill activities were delivered | 6 (100%) |
|  |  |  | N (%) of sessions where all required 3 fitness activities were delivered | 6 (100%) |
| Session 7 (Dad and Youngster session) | 4 | 6 | The fathers were engaged in the 'Welcome session' | 4.2 (0.8) |
|  |  |  | The youngsters were engaged in the 'Welcome session' | 4.5 (0.5) |
|  |  |  | The dads enjoyed the practical session | 4.5 (0.5) |
|  |  |  | The youngsters enjoyed the practical session | 4.5 (0.5) |
|  |  |  | N (%) of sessions where all required 4 rough and tumble activities were delivered | 5 (83%) |
|  |  |  | N (%) of sessions where all required 4 fundamental movement skill activities were delivered | 3 (75%) |
|  |  |  | N (%) of sessions where all required 3 fitness activities were delivered | 6 (100%) |
| Session 8 (Dad and Youngster session) | 4 | 6 | The fathers were engaged in the 'Welcome session' | 4.2 (0.8) |
|  |  |  | The youngsters were engaged in the 'Welcome session' | 4.7 (0.5) |
|  |  |  | The fathers and youngsters found the RTP "Youngsters choice" cards easy to follow | 4.7 (0.5) |
|  |  |  | The fathers and youngsters found the FMS "Youngsters choice" cards easy to follow | 4.7 (0.5) |
|  |  |  | The dads enjoyed the practical session | 4.5 (0.5) |
|  |  |  | The youngsters enjoyed the practical session | 5.0 (0.0) |
|  |  |  | N (%) of sessions where all required 3 fitness activities were delivered | 6 (100%) |
| Overall dad and youngster sessions | 4 | 48 | The fathers were engaged in the 'Welcome session' | 4.3 (0.6) |
|  |  |  | The youngsters were engaged in the 'Welcome session' | 4.5 (0.6) |
|  |  |  | The dads enjoyed the practical session | 4.7 (0.5) |
|  |  |  | The youngsters enjoyed the practical session | 4.7 (0.4) |
|  |  |  | N (%) of sessions where all required rough and tumble activities were delivered^¥^ | 40 (95%) |
|  |  |  | N (%) of sessions where all required fundamental movement skill activities were delivered^¥^ | 39 (93%) |
|  |  |  | N (%) of sessions where all required fitness activities were delivered | 43 (90%) |

*1= strongly disagree; 2= disagree; 3= neutral; 4= agree; 5= strongly agree.

^≠^ some facilitators delivered multiple sessions at different times. To note: participants were split across 3 programs running concurrently/staggered across the same day.

^¥^ Proportion is for 42 sessions as did not deliver in week 8 – instead youngster chose which RTP or FMS to complete

**References**

1. Tabachnic BG FL. Using Multivariate Statistic. 3rd ed. Boston, MA: Allyn & Bacon; 2001.
